# Development and validation of a scale to measure the care needs of Crohn’s disease patients: a mixed-methods study

**DOI:** 10.1186/s12912-024-02131-4

**Published:** 2024-07-10

**Authors:** Danlei Chen, Qing Liu, Zhihui Yu, Ting Pan, Ailing Zhang, Yan Chen, Fang Kong, ChengLiang Ding

**Affiliations:** 1 Nursing, Nanjing University of Chinese Medicine, Nanjing, 210023 China; 2 Department of Nursing, Nanjing Hospital, Affiliated to Nanjing University of Chinese Medicine (Nanjing Second Hospital), Nanjing, 210003 China; 3 School of Elderly Care Services and Management, Nanjing University of Chinese Medicine, Nanjing, 210023 China; 4 Nanjing Hospital Affiliated to Nanjing Medical University (Nanjing First Hospital), Nanjing, 210003 China; 5 Digestive Disease Treatment Center, Nanjing Hospital, Affiliated to Nanjing University of Chinese Medicine (Nanjing Second Hospital), Nanjing, 210003 China

**Keywords:** Crohn’s disease, Care needs, Supportive care, Scale

## Abstract

**Background:**

Crohn’s disease (CD) patients require varying levels of supportive care. In order to facilitate caregivers and nurses in precisely evaluating the caregiving requirements of these patients, we developed the CD-specific Care Needs Scale (CD-CNS).

**Methods:**

This study employed a mixed-methods approach, integrating qualitative and quantitative methodologies. The initial items of the scale were developed through qualitative interviews, Delphi expert consultation, and literature review, while the final items were refined through clinical testing. Qualitative interviews were conducted based on the supportive care needs framework and Maslow’s hierarchy of needs, and scale items were constructed through a literature search and qualitative interviews. The initial version of the scale with 45 items was obtained after the items were verified and modified by expert consultation. A total of 250 CD patients admitted to the gastroenterology department of a hospital in China were selected for verification of the initial version of the scale. A self-designed general questionnaire was used to obtain patients’ medical history and sociodemographic data, and the Chinese version of the Inflammatory Bowel Disease Questionnaire (IBDQ) was used as the criterion. Exploratory factor analysis (EFA) was performed on the CD-CNS to evaluate the dimensions, factor structure, reliability, criterion validity, and construct validity.

**Results:**

EFA identified 5 dimensions and retained 27 items with strong internal consistency reliability (α = 0.940). The Cronbach’s α coefficients for each dimension ranged from 0.824 to 0.921. Criterion validity was assessed using Spearman’s coefficient, which demonstrated a significant correlation with the IBDQ *(P* < 0.050). The test-retest reliability for each dimension after two weeks ranged from 0.655 to 0.895.

**Conclusions:**

We developed and validated a new scale that can be used to assess the care needs of CD patients. This new tool can guide the specific supportive care of CD patients.

**Trial registration:**

This study was reviewed and approved by the Ethics Committee of the Second Hospital of Nanjing (2021-LS-ky-022). The study was duly registered and approved online through the Trial Center of the Second Hospital of Nanjing in 2021. Confidentiality was ensured by anonymizing all the data. The entire study process was conducted under the supervision of the Ethics Committee of Nanjing Second Hospital. Informed consent was obtained from the patients, and each patient volunteered and agreed to participate.

**Supplementary Information:**

The online version contains supplementary material available at 10.1186/s12912-024-02131-4.

## Introduction

Crohn’s disease (CD) is a subtype of inflammatory bowel disease (IBD) [1] and is a chronic nonspecific intestinal inflammatory disease with unclear etiology and pathogenesis [2]. Currently, the number of CD patients in Asia is gradually increasing [3]. According to the predictions of the China Disease Prevention and Control Center (CDC), the number of IBD cases is expected to exceed 1.5 million by 2025. Although the incidence of CD is increasing, medical services related to CD are still imperfect in some areas, especially in underdeveloped areas [3].

At present, there is no specific drug available for curing this disease and clinical symptom management relies on long-term medication and dietary restrictions [4]. For certain patients, pharmacological interventions prove less than ideal, exposing them to recurrent and unclearly triggered episodes [5]. Emergency surgery is common, with studies indicating that at least half of patients require one or more surgical interventions [4]. Frequent bowel resections and prolonged stomas contribute to dysfunction [5]. While enteral nutrition improves nutritional status, prolonged tube feeding, whether in a hospital or home setting, not only increases the difficulty of self-management for patients [6], but also imposes significant economic burdens [7, 8]. Medications and surgeries provide only temporary relief of symptoms, necessitating continuous monitoring of the condition and prompting patients to seek timely medical attention [9]. Throughout the protracted course of the disease, patients have diverse needs in terms of medical, social support, and psychological aspects [10].

The concept of Supportive Care Needs (SCN) dates back to 1908 [11]. Currently, clinically recognized needs are defined by Dr. Fitch as supportive care, encompassing essential services and assistance throughout the disease treatment process, including survival, relief, and comfort. This involves addressing patients’ physiological, informational, emotional, psychological, and social needs. Despite exhibiting relatively high self-management capabilities [7], CD patients still necessitate support and care from caregivers, society, and the healthcare system. According to the results of studies designed to explore the needs of IBD patients during the healthcare-seeking process, patients require support from physicians to ensure adequate access to information about their conditions, medications, and diet, however, these forms of support are frequently overlooked in reality [12, 13]. These studies [12, 13] underscore the importance of personalized supportive care.

From a nursing perspective, the foundation of individualized supportive care necessitates an accurate assessment of each patient’s needs [13]. This ensures that caregivers, constrained by limited resources and personnel, focus on addressing patients’ primary concerns and relinquish non-condemned tasks. In the realm of social and family care, resource and personnel limitations compel caregivers to prioritize patients’ main problems, and provide as much effective help as possible in their limited energy. Consequently, there is a pressing need to delve into the matter of needs assessment in supportive care activities. Having a tool that can measure the level and extent of patients’ care needs is essential for standardized care and support. Caregivers can efficiently assess patients’ care needs through scientific means, take targeted measures to help address urgent issues, and reduce the waste of resources and energy.

Current studies addressing the care needs of patients with CD typically rely on assessment tools such as Inflammatory Bowel Diseases Questionnaire (IBDQ) [14]. However, these instruments require further exploration in terms of their comprehensive evaluation of caregiving needs for Crohn’s disease patients. The currently widely used supportive care needs scales and their brief versions, applicable to cancer patients [15], include some items that do not align with the situation of CD patients. This misalignment hinders the achievement of a thorough and accurate assessment of the unique caregiving needs of CD patients. Consequently, this study aimed to develop a straightforward evaluation tool to assess patients’ specific care needs. We hope that our scale will provide clinical healthcare professionals with an improved foundation for developing comprehensive clinical care plans for CD patients.

## Methods

### Design

This research employed a mixed-methods approach, integrating both qualitative (i.e., the study to generate items) and quantitative methodologies (i.e., the study to validate the items). We used an exploratory sequential mixed methods design and systematically integrated the qualitative and quantitative findings [16]. Using the building approach to integrate data, where the collected qualitative data is used to guide the quantitative data collection. During the research process, investigator triangulation and construct validation effectively integrate qualitative and quantitative data, providing a more comprehensive and nuanced understanding, thereby enhancing the validity and credibility of the research findings [16, 17].

**Fig. 1:**
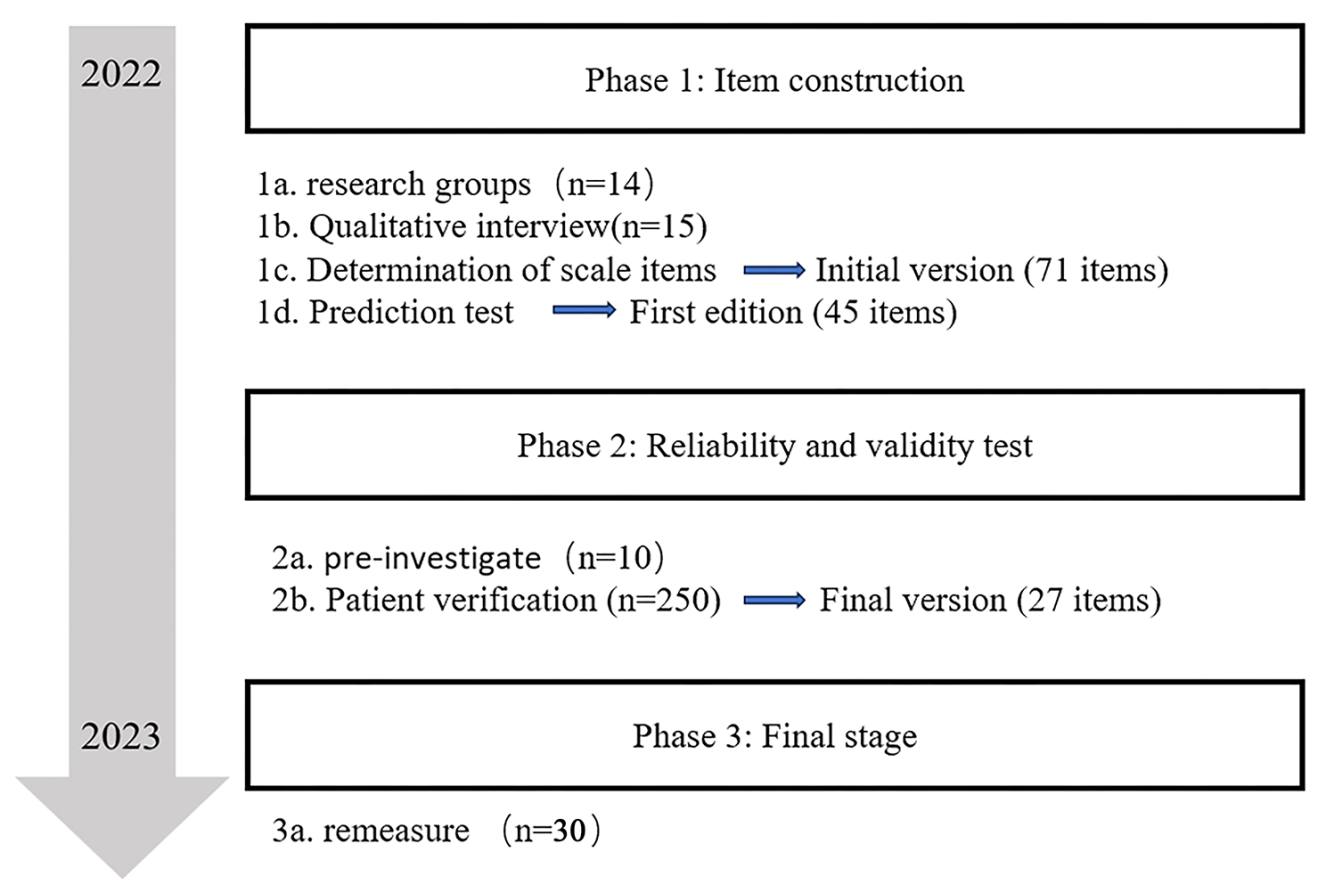
Scale preparation and testing process

A research team, initially constituted by 14 members, was assembled. The team included a master’s supervisor, the head of internal medicine, a specialist in Crohn’s disease, three chief nurses, one deputy chief nurse, a master of nursing, a medical doctor, a statistician, and four postgraduate nursing students. The research was conducted under the guidance of graduate students and supervisors, adhering to the standardized scale development process [15] as delineated in Fig. 1, encompassing three distinct stages: Item construction, Reliability and validity test, and Final stage. During the Item construction phase, qualitative interviews were conducted, and scale items were developed through the result of qualitative interviews, the Delphi method, and a comprehensive literature review. The scale underwent clinical testing in the Reliability and validity testing phase. Subsequently, in the Final stage, a retest was conducted to gather additional data on the scale’s performance.

### Development of the scale

#### Qualitative interview

Before formulating the scale, a preliminary qualitative study was undertaken. Drawing upon Dr. Fitch’s [11] defined theoretical framework of supportive care needs, we devised a semistructured interview outline tailored for qualitative research. The outline incorporated Maslow’s hierarchy of needs, encompassing physiological needs, security needs, love and belonging needs, respect needs, and self-realization needs, to supplement the framework. The interview outline as part of the scale development has not been published previously, and details are supplemented in Additional file (Additional file 1.docx). Employing interpretive phenomenological methods, qualitative interviews were conducted with three selected patients, and subsequent revisions led to the development of a formalized outline.

The qualitative interviews were guided by interpretive phenomenological methods, and patients diagnosed with CD from the Department of Gastroenterology at a hospital in Nanjing were chosen for interviews by combining objective sampling with theoretical sampling from September 2022 to October 2022. Eligible participants were required to be at least 18 years old, express readiness to engage in interviews, possess the capacity to provide in-person informed consent, and communicate effectively. Participants with other chronic diseases and who failed to provide complete responses to the interview outline were excluded.

The interviews were orchestrated by the head nurse of the department, with one nursing graduate student posing questions in line with the interview outline, while two other nursing graduate students diligently documented the proceedings. Prior to the interviews, all researchers underwent comprehensive training, encompassing linguistic familiarity and familiarity with the interview structure. The interviews were conducted in the classroom of the department. Face-to-face interviews were completed to avoid the presence and influence of other patients. The interviews were meticulously recorded through both notes and audio recordings, with explicit consent from each patient. Each interview lasted between 15 and 40 min. The recording materials were transcribed into written data within 24 h after the interview and uploaded to the hospital system for storage.

According to the principle of data saturation as the sample size in qualitative research, an interview should be terminated when no new themes or sub-themes appeared [18]. After 15 successful and comprehensive interviews, the researcher discerned the absence of novel themes. Following group discussions with experts, the research data were deemed complete, culminating in the cessation of further interviews. A total of 15 participants were involved in the study. The method of analyzing phenomenological data from Colaizzi [18] was used to analyze the data. The Colaizzi method provides a clear set of steps and framework that aids researchers in conducting structured data analysis within interpretive phenomenological studies. Through systematic procedures, researchers can extract and interpret key themes and concepts from participants’ descriptions, uncovering underlying meanings. The method emphasizes maintaining the originality and completeness of the data throughout the analysis process. This approach helps ensure the reliability and validity of the research, making the findings more persuasive and credible [18].

#### Determination of scale items

The items of the scale were determined mainly based on the results of qualitative research. According to the theme and sub-theme of qualitative research, members of the Group listed the relevant items. Two researchers systematically searched the database for the needs of CD patients from both Chinese domestic databases, including Wan Fang Data, CNKI, CBM, VIP, and international databases such as PubMed, Embase, The Cochrane Library, Web of Science, and CINAHL. The search period encompasses the inception of these databases up to January 6, 2023. Search terms consist of “Inflammatory Bowel Diseases”, “Crohn Disease”, “Needs Assessment”, “Demands”, “Needs”, and “Supportive Care Needs”. The included articles encompassed various research methodologies, such as qualitative studies, scales, interventions, systematic reviews, and expert consensus, all pertaining to CD patient care needs. After the literature was screened, the team members selected valuable content to supplement the items of the scale and developed the initial version of the scale, which included 94 items. The initial version of the CD-CNS, with 71 items, was developed by revising the original version and deleting unnecessary versions.

We extended the invitations to experts specializing in CD in China to participate in online Delphi expert consultations. We invited 12 experts to participate in the consultation, and 11 experts completed two rounds of consultations. The experts who participated in the expert consultation came from 6 regions of China, and they had certain experience in clinical Crohn’s disease or digestive disease nursing, treatment, education and other fields, with certain authority and professionalism. These experts assessed the significance and relevance of the scale items. Referring to established criteria outlined in previous studies [19]: (1) were engaged in or research in the field of Crohn’s disease treatment, nursing, scientific research, education and other fields for more than 5 years; (2) had a bachelor’s degree and intermediate professional titles or above; (3) were familiar with Crohn’s disease care-related content or relevant scale development experience; and (4) were willing to participate in two rounds of Delphi consultation. Experts were excluded if they failed to receive replies or did not return the questionnaire within two weeks of email inquiry. The consultation questionnaire, comprising a list of scale items for consultation and a collection of experts’ general information, was dispatched to each expert via email. Experts evaluated the importance of each item and provided feedback on their familiarity with the indicators and the basis for their judgment. Content modifications, additions, or deletions were implemented based on expert opinions. Ultimately, a refined 45-item version of the scale was derived.

#### Scale scoring method

The CD-CNS has five options for each question—no need, satisfied need, low need, moderate need, and high which are scored as 1, 2, 3, 4, and 5, respectively, with a score of approximately high indicating a greater degree of need for help.

#### Prediction test

Prior to the formal assessment of the revised scale, we conducted a preliminary investigation involving 10 patients to evaluate the clinical test version of the CD-CNS. Patients were queried about the scale’s reasonability and the clarity of its language. Subsequent adjustments to the scale’s language were made based on the patients’ feedback and opinions. The average completion time for the test was 5.8 ± 2.9 min. It is important to note that these patients were not included in the formal studies.

### Reliability and validity test of the scale

#### Research object and investigation methods

The study involved Grade III and Grade A hospitals in Nanjing, with participants consisting of patients admitted to the inflammatory bowel disease specialist in the gastroenterology department of these hospitals from May 2023 to June 2023. The head nurse explained the purpose, significance, and risks of the study to the patients and obtained their consent to participate in the study. The inclusion criteria were as follows: (1) patients clinically diagnosed with CD; (2) aged ≥ 18 years; (3) had clear awareness and ability to communicate in Mandarin; (4) were able to fill out questionnaires; and (5) were willing to cooperate with this study. The exclusion criteria for patients were as follows: (1) had a mental illness, cognitive impairment or other serious physical diseases; and (2) had severe emotional fluctuations during the questionnaire collection process. According to the factor analysis, the sample size should not be less than 5 to 10 times the number of items [20], and the formal test version of the scale contains a total of 45 items. Considering a 10% sample attrition, 250 patients are expected to be included in the survey. We invited 253 patients to participate in the validation, and ultimately, 250 patients’ questionnaires were deemed valid and included in the study. Two weeks later, 30 patients participated in the retest.

A nursing graduate student informed the patients about the purpose and significance of the study, and informed consent was obtained from the patients. A graduate student distributed paper questionnaires to the patients, and each questionnaire was verified by two researchers on the spot. The patient obtained evidence for doubtful answers on the spot and received valid questionnaires. Researchers received training in questionnaire distribution prior to the distribution of the questionnaire.

Questionnaires were disseminated either through social software or in person, and all patients were mandated to complete the questionnaires on the same day. Those who failed to submit the questionnaire within the specified timeframe or did not complete it were excluded from the analysis.

#### Data collection tools

The research tools employed in this study comprised the researchers’ designed general information questionnaire, the clinical test version of the CD-CNS, and the Chinese version of IBDQ [21]. The general information questionnaire aimed to gather the patient’s health status and medical details, encompassing gender, age, disease duration, marital status, occupation, residence, medical treatment method, caregiver, disease status, surgical history, nasogastric tube, stoma, abdominal drainage tube, and dietary pattern. Additional file provides both the general information questionnaire and the clinical test version of the CD-CNS (Additional file 2.docx).

The Inflammatory Bowel Diseases Questionnaire (IBDQ) was developed by Dr. Irvine [22] in 1989 to assess the quality of life in patients with inflammatory bowel disease. This scale comprises four dimensions and a total of 32 questions, evaluating systemic symptoms, intestinal symptoms, social functioning, and emotional functioning. The scale’s total score is 224, with higher scores indicating a better quality of life for patients with inflammatory bowel disease. The Chinese version of IBDQ was used in this study, it was translated by Zhou^2021^ and the Cronbach’s α coefficient of the scale in China was 0.949.

#### Scale evaluation

In qualitative research, triangulation methods are used to ensure that the findings accurately reflect the participants’ experiences and perspectives, thereby ensuring credibility. Transferability is achieved through a detailed description of the sample characteristics and research context [23]. Dependability is maintained by using clear and consistent research methods. Confirmability is ensured by keeping detailed records and documentation [24]. These credibility standards help ensure the rigor and reliability of qualitative research.

The content validity of the scale was evaluated using the Delphi method, with further validation through patient pre-testing and literature review. The scale’s validity was assessed through construct validity and criterion validity. The reliability of the scale was evaluated using Cronbach’s alpha coefficient and test-retest reliability.

#### Data analysis

The collected data were analyzed using SPSS 26.0. Descriptive statistics were employed to analyze participant characteristics, with count data presented as frequency and percentage, and normally distributed data presented as mean and standard deviation. The outcomes of expert consultation were conveyed through indicators such as the degree of authority, positive coefficient of experts, and the coefficient of variation of Kendall’s harmony coefficient [21]. Scale refinement involved modifications, deletions, or additions based on a comprehensive assessment considering the concentration and coordination of expert opinions, as well as expert modification suggestions.

Item analysis employed the critical ratio, correlation coefficient analysis, and Cronbach’s α. The main purpose of item analysis was to explore the difference between subjects with high and low scores on each item or to conduct a homogeneity test between items to judge the accuracy and reliability of the scale. Item retention was based on the critical ratio value and the homogeneity test. The critical ratio (CR) indicates that an item can indicate the response degree of a different subject; a CR is required to reach the level of significance (*P* < 0.050), and the statistical value should not be less than 0.300 [24]. The homogeneity test requires that the correlation coefficient between items and the total score and the total correlation coefficient of corrected items are not less than 0.400 [25].

Validity analysis encompassed content validity, construct validity, and criterion validity. Exploratory factor analysis (EFA) was used to analyze the construct validity, and the Keizer-Meyer-Olkin (KMO) measure of sampling adequacy and Bartlett’s sphere test were used to determine whether the data were suitable for EFA [26]. EFA requires the KMO value > 0.600 and a Bartlett’s test with a significance level of *P* < 0.050 [26]. Principal component analysis was used to carry out the varimax rotation. Entries with a loading factor < 0.4, entries with a load on both factors and match differences less than 0.1 were excluded [27] and the factor analysis was rerun each time an entry was deleted. Criterion validity reflects the degree of correlation between the scale measurement and the criterion measuremen [28]. Criterion validity was assessed through the examination of the correlation coefficient between the CD-CNS and the Chinese version of IBDQ.

In terms of reliability testing, Cronbach’s α was utilized to assess the reliability of both the overall scale and each dimension, while test-retest reliability was calculated two weeks after the initial test.

#### Rigour

To ensure credibility and reduce any possibility of bias in the researcher’s interpretation of the data, transcripts were returned to and approved by the participants after the qualitative study to ensure that they recognized these experiences as their own [12]. Qualitative research and clinical test results were analyzed by professional analysts to ensure the reliability of the results.

## Results

### Qualitative interview

After 15 successful and comprehensive interviews, the researcher discerned the absence of novel themes. Following group discussions with experts, the research data were deemed complete, culminating in the cessation of further interviews. A total of 6 themes and 26 subthemes were extracted. These themes included treatment-related needs, life needs, physical needs, psychological needs, external support needs, and growth and benefits.

### Expert enquiry

Eleven experts completed 2 rounds of consultation, and the age of the experts was 31–47 (38.46$\pm$4.07) years. The length of professional experience ranged from 6 to 28 (14.64$\pm$6.31) years; 1 (9.09%) had a doctor’s degree, 2 (18.18%) had a master’s degree, and 8 (72.72%) had a bachelor’s degree. There was 1 (9.09%) college teacher and 10 (90.90%) clinical nurses. The questionnaire recovery rates were 91.7% and 100%, respectively. In the first round, the expert authority coefficient is 0.827, the Kendall harmony coefficient is 0.257, ${\chi }^{2}=$is 197.706, and *P* is $<$ 0.010. The revised scale has 5 dimensions and 54 items. In the second round, the expert authority coefficient was 0.824, the Kendall harmony coefficient was 0.261, ${\chi }^{2}=$152.019, and *P*$<$ 0.010. The revised scale has 5 dimensions and 45 items.

### Patient general characteristics

In this study, questionnaires were distributed to 253 patients. However, three participants submitted identical answers to all questions but declined to provide explanations, leading to their exclusion. Consequently, the final analysis involved 250 patients (Table 1), comprising 182 males (72.8%) and 68 females (27.2%). The average age was 37.7$\pm$12.6 years, and the mean disease duration was 6.8$\pm$5.4 years.

**Table 1:** Participant characteristics

Characteristics	Categories	Total %(*n*)
Stage	Acute episode	23.6% (59)
	Remission	76.4% (191)
Disease State	Surgery	73.2% (183)
	Complication	39.2% (98)
Tube	Nasal feeding tube	34.8% (87)
	Abdominal drainage tube	6.0% (15)
	Stoma	2.4%( 6)
Diet	Normal diet	42.0% (105)
	Enteral nutrition	28.0% (70)
	Normal diet/Enteral nutrition	30.0% (75)
Residence	Rural area	44.0% (110)
	City	56.0% (140)
Employment status	Students	10.8% (27)
	Employment	72.8% (182)
	Unemployment	16.4% (41)
Medical expense	Medical insurance	91.2% (228)
	Self-paying	8.8% (22)
Marital status	Married	61.2% (153)
	Unmarried	38.8% (97)
Caregiver	Parents	42.4% (106)
	Spouse	36.8% (92)
	Oneself	12.0% (30)
	Other	8.8% (22)

### Item analysis

The results of the item analysis showed that the CR values for all 45 items exceeded 3.0, and the significance level (*P*) was less than 0.050, meeting the inclusion criteria and consequently retained. The Cronbach’s α of the clinical test version of the CD-CNS was 0.965, and the Cronbach’s α of deleting this item was 0.964 ~ 0.965.

### Content validity

The scale of content validity index (S-CVI) of the CD-CNS was 0.970, and the item of content validity index (I-CVI) was 0.818 to 1.000.

### Construct validity

The KMO value was 0.937, and the results of Bartlett’s test were ${\chi }^{2}=$152.019, *P*$<$0.010; therefore, the sample was suitable for conducting EFA. A total of 8 factors with eigenvalues greater than 1 were extracted, and the cumulative variance contribution rate was 72.053%. After seven replicates, five common factors were obtained, for a cumulative variance contribution rate of 66.084% (Table 2). After discussion by experts in the group, we named them Medical Needs, Information Needs, Physiological Needs, Psychological Needs and External Support Needs.

**Table 2:** Factor loadings from exploratory factor analysis

Item	Physiological needs	Psychological needs	Medical needs	Information needs	External support needs
1 I need some disease experts and expert information			0.573		
2 I need local treatment services for Crohn’s disease			0.643		
3 I need services related to specialist and hospital appointments			0.791		
4 I hope the doctor can simple, detailed, honest to my illness			0.735		
5 I hope to be given enough opportunity and time to communicate with doctors			0.757		
6 I need accurate disease diagnosis			0.746		
11 I need to be provided with the latest disease research				0.644	
12 I need to be given some information about drug				0.557	
14 I need to be given some information about diet				0.804	
15 I need to be given some information about sports				0.796	
18 I need to be given some disease management plan				0.560	
23 I need help to deal with abdominal pain	0.780				
24 I need help to deal with weakness	0.756				
25 I need help to deal with weight loss	0.654				
26 I need help to deal with diarrhea	0.740				
27 I need help to deal with insomnia	0.589				
28 I need help to deal with discomfort caused by pipes	0.542				
29 I need help to deal with weakness desire to diet	0.569				
36 I need professional psychological counseling		0.766			
37 I need help to cope with anxiety		0.844			
38 I need help to cope with depression		0.866			
39 I need help to cope with loneliness		0.828			
41 I need help to cope with stigma		0.597			
31 I need extra supplies					0.788
34 I need to be provided home care services					0.660
42 I need additional employment benefits					0.668
43 I require additional financial assistance					0.771

Only factor loading values greater than 0.4 are shown

### Criterion validity

For criterion validity, the correlation between the CD-CNS score and the Chinese version of the IBDQ was *r*=$-$0.458 (*P* < 0.050).

### Reliability

The Cronbach’s α of the CD-CNS was 0.940, and the Cronbach’s α coefficients and test-retest reliability for each dimension of the scale are shown in Table 3.

**Table 3:** Reliability of each dimension of the scale

Reliability	Dimension one	Dimension two	Dimension three	Dimension four	Dimension five
Cronbach’s α	0.862	0.836	0.891	0.921	0.824
Test-retest Reliability	0.895	0.824	0.883	0.655	0.829

## Discussion

We modified the CD-CNS through a literature search, qualitative interviews, the Delphi method and expert verification. According to the results of clinical test, the scale has good content validity and structural validity [26], and the scale score was negatively correlated with the total score on the Chinese version of the IBDQ [28]. However, the retest reliability of the psychological dimension is relatively low. Team members posit that emotional fluctuations in Crohn’s disease patients are influenced by the course of the disease [29]. Moreover, they observe that the same patient may have varying needs for emotional regulation at different times. Substantiating this hypothesis may necessitate repeated measurements over distinct time intervals in the future. Previous research [30] has found that as the number of tests and items increases, participant engagement may decline, and respondent fatigue may occur due to the length of the scale. In our scale, the psychological dimension is positioned in the latter half, which might be a factor contributing to its lower reliability. Despite the lower retest reliability in the psychological dimension, the overall reliability of the scale is high. The findings indicate that the scale demonstrates scientific validity concerning its theoretical foundation, structure, and content.

The CD-CNS assesses 27 items in 5 dimensions, namely, medical treatment, information, physical, psychological and external support. The dimension of medical needs is mainly used to assess patients’ needs related to disease experts, diagnosis, and medical environment (including medical conditions and humanities) during the process of medical treatment. In developing regions and countries, CD remains largely overlooked by the public [31]. Limitations in disease diagnosis and professional expertise often lead to misdiagnosis and incorrect treatment [32]. Consequently, CD patients require greater medical assistance, including faster access to treatment, more comprehensive services from physicians, and accurate treatment plans. The Information Needs dimension can assess the information needs of patients, including the need for research, drugs, diet and exercise. Although CD is currently incurable, increasing research and the development of new medications provide effective means for disease control. Notably, many previous studies [33, 34] have confirmed that healthcare professionals often neglect to provide patients with information on new research developments and medications, leaving patients’ needs for drug and treatment-related information unmet. As patients become more health-conscious, they increasingly value information related to their overall well-being, including dietary and exercise guidance. Providing such information will be crucial for future patient care. Physiological needs can assess patients’ need for help in addressing common disease symptoms and physical discomfort. Patients’ physiological needs are primarily related to their physical symptoms and stem from the desire to alleviate various symptoms. For CD patients, they often cannot control symptoms such as diarrhea, weight loss, and weakness on their own and struggle to cope with the discomfort caused by various complications [35, 36]. Therefore, they require assistance from professional healthcare institutions. Psychological needs can assess the needs of patients to cope with negative emotions. Crohn’s disease patients face psychological issues from multiple sources, experiencing high levels of psychological stress and various negative emotions that severely impact their quality of life [37, 38]. While some patients can alleviate stress through self-regulation [39], many choose to endure discomfort on their own due to previous unsuccessful attempts to seek help. Assessing patients’ psychological needs is crucial. Although research indicates that psychological interventions can effectively alleviate negative emotions [40], our survey found that most patients prefer self-coping over professional psychological interventions. This suggests that future psychological interventions should focus on enhancing patients’ self-regulation abilities. Patients’ needs for outside help can be assessed through the use of external support, including daily care, materials and preferential life policies provided by public welfare organizations and groups, communities, hospitals, and other policies. The increasing incidence of CD has already placed a burden on the healthcare systems of some developed countries [41]. Therefore, supporting the care of CD patients requires broader efforts, including contributions from charitable organizations and community groups. The final version of the scale is provided in Additional file 2.

As an incurable lifelong disease, patients with Crohn’s disease (CD) exhibit various needs throughout the treatment process [42]. Effectively assessing patients’ care requirements and providing personalized supportive care can not only significantly enhance their quality of life but also efficiently reduce the waste of healthcare resources [43, 44]. CD-CNS, serving as an assessment tool, contributes to evaluating the current status of various needs in Crohn’s disease patients. The scale can assess the supportive care needs of patients with Crohn’s disease, so that caregivers can comprehensively, individually and standardized assess the care needs of patients [45]. The collection of patients’ needs helps to develop care plans and nursing services that meet the needs of patients.

### Limitations

The current study is significant because it developed a reliable and valid tool to evaluate the care needs of CD patients. Due to the lack of specialized hospitals that can cure CD in China, as one of the most influential hospitals for treating this disease, we were fortunate to collect CD patients from all over China. A total of 265 patients were included in this study. Patients were from more than 40 regions in 11 provinces of China and were adequately representative. However, the scale has not been verified in other languages or groups, which is a limitation of this study. The test-retest analysis in this study was only conducted after two weeks. Although two weeks was appropriate for some studies [46], this may be a factor leading to the low reliability of the retest due to the large differences in different stages of CD [47, 48]. In the future, it will be necessary to expand the retest interval to prove the stability of the scale.

## Conclusion

We developed and validated a scale that can be used to assess the care needs of patients with CD. The development of the scale was based on a standardized scale development process, which included qualitative interviews, a literature search, expert letter inquiries, expert verification and patient verification. The CD-CNS has 5 dimensions and a total of 27 items; it has good reliability and validity and a significant negative correlation with the IBDQ score. The CD-CNS can be used to assess patients’ medical, informational, physical, psychological and external support needs and to provide tools and ideas for the development of care programs and nursing services in line with patients, which can be used in clinical practice.

### Electronic supplementary material

Below is the link to the electronic supplementary material.

Supplementary Material 1

Supplementary Material 2

Supplementary Material 3

## Data Availability

All data is given in the paper, open and available.

## References

[1] ,NOD2 deficiency increases retrograde transport of secretory IgA complexes in Crohn’s disease. Nature communications, Veauthier B, Hornecker JR. (2018). Crohn’s Disease: Diagnosis and Management. American family physician, 98(11), 661–669. 30485038

[2] Ooi CJ, Makharia GK, Hilmi I (2016). Asia-Pacific consensus statements on Crohn’s disease. Part 2: management. J Gastroenterol Hepatol.

[3] Baumgart DC (2009). The diagnosis and treatment of Crohn’s disease and ulcerative colitis. Dtsch Arztebl Int.

[4] Stöss C, Berlet M, Reischl S (2021). Crohn’s disease: a population-based study of surgery in the age of biological therapy. Int J Colorectal Dis.

[5] Adamina M, Bonovas S, Raine T (2020). ECCO Guidelines on therapeutics in Crohn’s Disease: Surgical Treatment. J Crohns Colitis.

[6] Trindade IA, Ferreira C, Pinto-Gouveia J (2016). Inflammatory bowel disease: the harmful mechanism of experiential avoidance for patients’ quality of life. J Health Psychol.

[7] Singh S, Murad MH, Fumery M (2021). Comparative efficacy and safety of biologic therapies for moderate-to-severe Crohn’s disease: a systematic review and network meta-analysis. Lancet Gastroenterol Hepatol.

[8] Barreiro-de Acosta M, Molero A, Artime E (2023). Epidemiological, clinical, patient-reported and economic Burden of Inflammatory Bowel Disease (Ulcerative colitis and Crohn’s disease) in Spain: a systematic review. Adv Ther.

[9] Peyrin-Biroulet L, Ghosh S, Lee SD (2023). Effect of risankizumab on health-related quality of life in patients with Crohn’s disease: results from phase 3 MOTIVATE, ADVANCE and FORTIFY clinical trials. Aliment Pharmacol Ther.

[10] Singh S, Boland BS, Jess T, Moore AA (2023). Management of inflammatory bowel diseases in older adults. Lancet Gastroenterol Hepatol.

[11] FITCH M I (2008). Supportive care framework[J]. Can Oncol Nurs J.

[12] Cho R, Wickert NM, Klassen AF (2018). Identifying needs in young adults with inflammatory bowel disease: a qualitative study. Gastroenterol Nursing: Official J Soc Gastroenterol Nurses Associates.

[13] di Giuseppe R, Plachta-Danielzik S, Mohl W (2021). Profile of patients with inflammatory bowel disease in conjunction with unmet needs and decision-making for choosing a new biologic therapy: a baseline analysis of the VEDOIBD-Study. Int J Colorectal Dis.

[14] Papamichael K, Afif W, Drobne D (2022). Therapeutic drug monitoring of biologics in inflammatory bowel disease: unmet needs and future perspectives. Lancet Gastroenterol Hepatol.

[15] Chunfeng LIU, Rong W, Xiaomei Z (2019). Preparation and validity of supportive care need scale for patients with cancerous wounds. Nurs Res.

[16] Shiyanbola OO, Rao D, Bolt D (2021). Using an exploratory sequential mixed methods design to adapt an illness perception Questionnaire for African americans with diabetes: the mixed data integration process. Health Psychol Behav Med.

[17] Hammerschmidt J, Manser T (2019). Nurses’ knowledge, behaviour and compliance concerning hand hygiene in nursing homes: a cross-sectional mixed-methods study. BMC Health Serv Res.

[18] Colaizzi P (1978). Psychological research as a phenomenologist views it[M]//Valle. King M.Existential phenomenological alternatives for psychology.

[19] BaoGuanJun L, Ye L, Yuanfei. Community elderly fall risk perception scale and test reliability and validity of [J]. J Nurs Sci.2022; ((24): 9–13.

[20] Wang Y, Li S, Gong J, Cao L (2022). Perceived stigma and self-efficacy of patients with inflammatory bowel Disease-Related Stoma in China: a cross-sectional study. Front Med.

[21] Zhou YX. Clinical application of the Chinese version of the inflammatory bowel disease quality of life questionnaire. Zhejiang University,2006. (in Chinese).

[22] Guyatt G, Mitchell A, Irvine EJ, Singer J, Williams N, Goodacre R, Tompkins C (1989). A new measure of health status for clinical trials in inflammatory bowel disease. Gastroenterology.

[23] Porter S (2007). Validity, trustworthiness and rigour: reasserting realism in qualitative research. J Adv Nurs.

[24] Au A, Lam WW, Kwong A (2011). Validation of the Chinese version of the short-form supportive care needs Survey Questionnaire (SCNS-SF34-C). Psycho-oncology.

[25] Luquiens A, Whalley D, Crawford SR (2015). Development of the Alcohol Quality of Life Scale (AQoLS): a new patient-reported outcome measure to assess health-related quality of life in alcohol use disorder. Qual life Research: Int J Qual life Aspects Treat care Rehabilitation.

[26] Li Y, Binqi Y, Huanhuan L. Reliability and validity of revised version of primary caregiver information needs scale for children with cancer. Mod Prev Med 2015,42(08):1422–5. (in Chinese).

[27] Gu Fangchen. Development and preliminary application of symptom cluster assessment scale for inflammatory bowel disease patients based on symptom experience model. Nanjing medical university. 2021. DOI: 10.27249 /, dc nki. Gnjyu. 2020.000149. (in Chinese).

[28] Niu Aifang F, Jufang Z. Ruili Localization of supportive care demand scale for caregivers of cancer patients. Chin J Nurs 2015,32(16):6–10. (in Chinese).

[29] Ghasemi Fard F, Mirzaei H, Hosseini SA (2021). Development and clinimetric assessment of a performance-based functional vision tool in visually impaired children. Front Pead.

[30] Yew SQ, Tan KA, Nazan AINM, Manaf RA (2023). Development and validation of a medication non-adherence scale for Malaysian hypertensive patients: a mixed-methods study. Environ Health Prev Med.

[31] Canavan C, Abrams KR, Hawthorne B, Drossman D, Mayberry JF (2006). Long-term prognosis in Crohn’s disease: factors that affect quality of life. Aliment Pharmacol Ther.

[32] Chen Y (2018). Perspectives of IBD China: is Crohn’s and Colitis Foundation Model a solution to Health Care issues for the country?. Inflamm Bowel Dis.

[33] Daher S, Khoury T, Benson A (2019). Inflammatory bowel disease patient profiles are related to specific information needs: a nationwide survey. World J Gastroenterol.

[34] Revés J, Ungaro RC, Torres J (2021). Unmet needs in inflammatory bowel disease. Curr Res Pharmacol Drug Discov.

[35] Rozich JJ, Holmer A, Singh S (2020). Effect of lifestyle factors on outcomes in patients with inflammatory Bowel diseases. Am J Gastroenterol.

[36] Vieujean S, Louis E (2023). Precision medicine and drug optimization in adult inflammatory bowel disease patients. Th Adv Gastroenterol.

[37] Schoefs E, Vermeire S, Ferrante M (2023). What are the unmet needs and most relevant Treatment outcomes according to patients with inflammatory bowel disease? A qualitative patient preference study. J Crohns Colitis.

[38] Zanoli L, Tuttolomondo A, Inserra G (2020). Anxiety, depression, chronic inflammation and aortic stiffness in Crohn’s disease: the brain–gut–vascular axis. J Hypertens.

[39] Lovén Wickman U, Yngman-Uhlin P, Hjortswang H (2016). Self-care among patients with inflammatory bowel disease: an interview study. Gastroenterol Nurs.

[40] Sinopoulou V, Gordon M, Akobeng AK (2021). Interventions for the management of abdominal pain in Crohn’s disease and inflammatory bowel disease. Cochrane Database Syst Rev.

[41] Buie MJ, Quan J, Windsor JW (2023). Global hospitalization trends for Crohn’s Disease and Ulcerative Colitis in the 21st Century: a systematic review with temporal analyses. Clin Gastroenterol Hepatol.

[42] Bisgaard TH, Allin KH, Keefer L (2022). Depression and anxiety in inflammatory bowel disease: epidemiology, mechanisms and treatment. Nat Rev Gastroenterol Hepatol.

[43] Fourie S, Jackson D, Aveyard H (2018). Living with inflammatory bowel disease: a review of qualitative research studies. Int J Nurs Stud.

[44] Danese S, Allez M, van Bodegraven AA et al. Unmet Medical needs in Ulcerative Colitis: an Expert Group Consensus. Digestive diseases (Basel, Switzerland).2019; 37(4), 266–83. 10.1159/000496739. 10.1159/000496739 30726845

[45] Hart NH, Crawford-Williams F, Crichton M (2022). Unmet supportive care needs of people with advanced cancer and their caregivers: a systematic scoping review. Crit Rev Oncol/Hematol.

[46] Mikocka-Walus A, Olive L, Skvarc D (2020). Expressive writing to combat distress associated with the COVID-19 pandemic in people with inflammatory bowel disease (WriteForIBD): a trial protocol. J Psychosom Res.

[47] Levine A, Wine E, Assa A (2019). Crohn’s Disease Exclusion Diet Plus partial Enteral Nutrition induces sustained remission in a Randomized Controlled Trial. Gastroenterology.

[48] Del Hoyo J, Nos P, Bastida G (2019). Telemonitoring of Crohn’s Disease and Ulcerative Colitis (TECCU): cost-effectiveness analysis. J Med Internet Res.

