# Supplementary material for: Development and validation of a scale to measure the care needs of Crohn’s disease patients: a mixed-methods study
Source: BMC Nurs. 2024 Jul 10;23:472. doi: 10.1186/s12912-024-02131-4 (PMC11234523; doi:10.1186/s12912-024-02131-4)
Supplement: Supplementary file 1 — Supplementary Material 1 [file 12912_2024_2131_MOESM1_ESM.docx]

**Interview outline**

1. What physical discomfort does Crohn's disease bring to you? How do you relieve these discomforts？

2. How did you feel when you were told you had the disease? What is the current view of Crohn's disease? (Disease, treatment)

3. What measures do you think can help you relieve your emotional distress?

4. What information do you think is important in the process of disease treatment, and what information do you want to know but can't get now?

5. What financial difficulties did you have during the treatment? What are your current economic needs? (Fundraising intention, government subsidy)

6. How does the disease affect your daily work and life? (Work, social, family)

7. What do you think of your illness from the perspective of others? Do you feel understood and accepted?

8. Do you feel that the people around you know about Crohn's disease? What kind of help do you hope the outside world (hospital and family) can provide you?

9. What are your current medical needs? (Experts, hospital selection) Are you satisfied with the hospital environment and attitude of care?

10. What are your views and plans on the future life of illness? (Marriage, childbirth, study and work)

11. What do you expect your life to be like? Is the current situation meeting your expectations?
