# Supplementary material for: Development and validation of a scale to measure the care needs of Crohn’s disease patients: a mixed-methods study
Source: BMC Nurs. 2024 Jul 10;23:472. doi: 10.1186/s12912-024-02131-4 (PMC11234523; doi:10.1186/s12912-024-02131-4)
Supplement: Supplementary file 2 — Supplementary Material 2 [file 12912_2024_2131_MOESM2_ESM.docx]

## General information questionnaire

| Name： | Gender: | | Age: | Time of diagnosis: |
| --- | --- | --- | --- | --- |
| Marital status: □ Married □ Unmarried □ Divorced □ Widowed □ Other | | | | |
| Fertility status: □ No □ Yes | | | | |
| Occupation: □ worker□ student □ unemployed | | | | |
| Education level: □ Primary school or below and illiterate □ primary school □ junior high school □ high school or college □ undergraduate □ graduate or above | | | | |
| Long-term residence: □ Rural □ urban | | | | |
| Forms of medical expenses: □ Self-paid □ Medical insurance □ public funded | | | | |
| Current work status: □ Full time work □ Part time work □ Sick leave □ No work | | | | |
| Family monthly income: □ < = 3000 □3001~6000 □6001~10000□>10000 | | | | |
| Annual disease expenditure: □ < = 5000 □5000~10000□>10000 | | | | |
| Family main source of income: □ own □ parents □ spouse □ children □ others | | | | |
| Primary Caregiver: □ Parents □ Spouses □ Children □ Other | | | | |
| Current disease status:  □ During attack □ During remission | | | Biologic agent use status:  □ Yes □ No | |
| Purpose of this admission: □ medication □ surgery □ review | | | | |
| Surgery: □ Yes □ No | | | Complications: □ Yes □ No | |
| Nasogastric tube:  □ Yes □ No | | Stoma:  □ yes □ No | | Abdominal double tube: □ yes □ no |
| Current diet: □ normal diet □ Restricted diet □ Total GI nutrition □ Supplementary GI nutrition | | | | |

## Clinical test version of the CD-CNS

This scale encompasses 27 items aimed at delineating your care requirements. Assess whether you necessitate assistance with the following issues over the past month based on your current circumstances. Each query featured five response options: no need, satisfied need, low need, moderate need, and high need, corresponding to a scoring system of 1, 2, 3, 4, and 5 points, respectively. Kindly choose the fitting option from the following:

1. I need some disease experts and expert information.
2. I need local treatment services for Crohn's disease.
3. I need services related to specialist and hospital appointments.
4. I hope the doctor can simple, detailed, honest to my illness.
5. I hope to be given enough opportunity and time to communicate with doctors.
6. I need accurate disease diagnosis.

7. I need to be provided with viable surgical and therapeutic options

8. I need to be informed about the possible future course of the disease

9. I need to be provided with treatment for adverse reactions and complications of the surgery

10. I need to know when I need to see a doctor or get a review

11. I need to be provided with the latest disease research

12. I need to be given some information about drug

13. I need to be able to be provided with a greater variety of enteral nutrition formulations

14 I need to be given some information about diet

15 I need to be given some information about sports

16. I need to be given guidance on nasal feeding self-care

17. I need to be provided with ostomy self-care guidance

18. I need to be given some disease management plan

19. I need to be able to be given guidance and advice on employment

20. I need someone to help me ease my family problems

21. I need help to cope with my change in lifestyle

22. I need help with the transition between family, work, and school

23. I need help to deal with abdominal pain

24. I need help to deal with weakness

25. I need help to deal with weight loss

26. I need help to deal with diarrhea

27. I need help to deal with insomnia

28. I need help to deal with discomfort caused by pipes

29. I need help to deal with weakness desire to diet

30. I need help from my family

31. I need extra supplies

32. I need doctors and nurses to have a better attitude

33. I need to be provided with better hospital, ward, and community environment

34. I need to be provided home care services

35. I need online medical service

36. I need professional psychological counseling

37. I need help to cope with anxiety

38. I need help to cope with depression

39. I need help to cope with loneliness

40. I need help with self-blame and guilt

41. I need help to cope with stigma

42. I need additional employment benefits

43. I require additional financial assistance

44. I hope to gain the respect of others

45. I need help to reduce my family's worries about me

## CD-CNS: CD-specific Care Needs Scale

This scale encompasses 27 items aimed at delineating your care requirements. Assess whether you necessitate assistance with the following issues over the past month based on your current circumstances. Each query featured five response options: no need, satisfied need, low need, moderate need, and high need, corresponding to a scoring system of 1, 2, 3, 4, and 5 points, respectively. Kindly choose the fitting option from the following:

1. I need some disease experts and expert information.

2. I need local treatment services for Crohn's disease.

3. I need services related to specialist and hospital appointments.

4. I hope the doctor can simple, detailed, honest to my illness.

5. I hope to be given enough opportunity and time to communicate with doctors.

6. I need accurate disease diagnosis.

1. I need some disease experts and expert information.
2. I need to be given some information about drug.
3. I need to be given some information about diet.
4. I need to be given some information about sports.
5. I need to be given some disease management plan.
6. I need help to deal with abdominal pain.
7. I need help to deal with weakness.
8. I need help to deal with weight loss.
9. I need help to deal with diarrhea.
10. I need help to deal with insomnia.
11. I need help to deal with discomfort caused by pipes.
12. I need help to deal with weakness desire to diet.
13. I need professional psychological counseling.
14. I need help to cope with anxiety.
15. I need help to cope with depression.
16. I need help to cope with loneliness.
17. I need help to cope with stigma.
18. I need extra supplies.
19. I need to be provided home care services.
20. I need additional employment benefits.
21. I require additional financial assistances.
